# The Relationships among Self-Worth Contingency on Others’ Approval, Appearance Comparisons on Facebook, and Adolescent Girls’ Body Esteem: A Cross-Cultural Study

**DOI:** 10.3390/ijerph18030901

**Published:** 2021-01-21

**Authors:** Michael Prieler, Jounghwa Choi, Hye Eun Lee

**Affiliations:** 1 Media School, Hallym University, Chuncheon 24252, Korea; prieler@hallym.ac.kr; 2 Department of Advertising & Public Relations, Hallym University, Chuncheon 24252, Korea; 3 School of Communication and Media, Ewha Womans University, Seoul 03760, Korea; hyeeunlee77@ewha.ac.kr

**Keywords:** social networking sites, female adolescents, body esteem, self-worth contingency on others’ approval, culture

## Abstract

The present study examined the relationship between appearance-related social comparison on social networking services (SNSs) and body esteem in a cross-cultural context (three European countries, i.e., Austria, Belgium, and Spain, versus one Asian country, i.e., South Korea). The role of self-worth contingency on others’ approval was considered to be a psychological and cultural factor. Utilizing a large-scale cross-national survey of early and middle adolescents in 2017, the responses of female adolescents (*N* = 981) were analyzed. The results generally support the findings from previous studies but also reveal cultural differences. Appearance comparison on Facebook negatively influenced girls’ body esteem in all European countries, but not in South Korea. Self-worth contingency on others’ approval negatively influenced girls’ body esteem across all four countries. Finally, a positive relationship between self-worth contingency on others’ approval and appearance comparison on Facebook was found in all European countries, but not among Korean girls. These findings suggest the importance of self-worth contingency on others’ approval and cultural contexts can be used to study the effects of body image-related SNS use.

## 1. Introduction

How teenaged girls appreciate their own bodies is an important source of their psychological and emotional well-being [1]. A number of studies have reported that young women tend to have a more negative body image than young men [2]. While exposure to idealized body images on mass media has been identified as a primary cause of body image disturbance among adolescent girls [3], social networking sites (SNSs) are receiving increasing attention from scholars, as they are becoming a primary media source for adolescents across the globe [4,5].

Perloff argued that SNS use can be detrimental to girls’ body image because SNSs can provide a greater opportunity for negative social comparison than traditional media [6]. It has been reported that people tend to present themselves on SNSs in overly positive ways [7] and, thus, their self-presentation on SNSs is positively skewed [8,9]. As a result, upward body image comparison may easily occur on SNSs [10]. As women are more likely than men to use SNSs for social comparison [11], such negative body image comparisons on SNSs are expected to negatively affect teenaged girls in particular.

Several studies have shown that SNS use—and, in particular, appearance-oriented use, such as posting or viewing photographs—contributes to body image-related outcomes, such as internalization of the thin ideal in females and body dissatisfaction [12,13]. However, few studies have explored the psychological factors that drive such body image-related SNS behaviors. Understanding such psychological factors is important because it can provide insight for designing body image intervention programs and social media literacy programs. Additionally, given that the majority of previous studies have been conducted in Western cultures, cross-cultural studies will be necessary to fully understand the role of SNSs in girls’ body image disturbances. Although some effects of SNSs may be universal, the roles that SNSs play and how people use SNSs in different societies may vary depending on cultures [14,15]. Such cultural differences may, in turn, affect girls’ use of SNSs and its subsequent consequences.

To address this issue, the present study explores how appearance-related social comparison on SNSs is related to body esteem in a cross-cultural context (three European countries, i.e., Austria, Belgium, and Spain, versus one Asian country, i.e., South Korea). In carrying out this comparison, we consider the role of self-worth contingency on others’ approval as a psychological factor that potentially drives girls’ social comparison behavior on SNSs. Self-worth contingency on others’ approval, which is one of the domains of self-worth contingency [16], is expected to be culturally sensitive. Based on Markus and Kitayama [17], among Koreans who are likely to have lower independent self-construal compared to those in European countries, others’ approval contingent self-worth may play a more prominent role because others’ views are integral to shaping one’s own self-concept. Although several studies have explored the relationship between appearance comparison and self-worth contingency [18], this relationship has rarely been discussed in the context of SNS use.

By exploring the relationship of self-worth contingency on others’ approval with appearance comparisons on SNSs and body esteem among teenaged girls in a cross-cultural context, this study aims to provide practical implications for body image intervention programs for girls in addition to obtaining more generalized findings on the roles of social media among adolescent girls across diverse cultures.

### 1.1. Social Media, Appearance Comparison, and Body Esteem

Numerous studies addressing media effects on body image have been conducted throughout the last several decades. These studies acknowledge the central importance of the media in imparting unrealistic images of female beauty [6]. Several correlational and experimental studies have found positive correlations between mass media exposure and negative body image and related outcomes [19,20,21,22,23,24,25].

In the last few years, SNSs have received increasing academic attention from body image researchers [12,26]. SNS refers to a group of Internet-based applications that “allow individuals to present themselves, articulate their social networks, and establish or maintain connections with others” [27] (p. 1143). Due to certain features, SNSs have important implications for body image concerns. On SNSs, users can create and post their own content in rich modalities, and self-disclosure plays a prominent role in this process. Because users tend to present themselves on SNSs in overly positive ways [7], and as the media of one’s peers, SNSs may allow for easy and frequent access to attractive peers [6]. As adolescent girls are highly sensitive to peer comparison [28], and upward comparison with attractive peers (compared to attractive advertising models) can result in a more negative evaluation of one’s own body [29], such exposure on SNSs may prompt body dissatisfaction.

Some of the empirical studies concerning SNSs support this prediction, suggesting that general SNS use has a positive association with various body image-related outcomes, such as appearance comparison, internalization of the thin ideal in females, and body dissatisfaction [12]. However, other studies found that general SNS use does not correlate with body dissatisfaction and related outcomes [30,31,32,33]. Instead, correlations were found when users engaged in appearance-oriented SNS use, such as posting or viewing photographs [34,35]. These findings indicate that the use of appearance-oriented SNSs, rather than SNS use itself, is a stronger and clearer predictor of body dissatisfaction and related outcomes.

As a variable that captures appearance-oriented SNS use, we focused on appearance-related social comparison behavior on SNSs. While individuals have a natural drive to compare themselves to others for self-evaluation purposes [36], appearance comparison has been found to have negative consequences in terms of women’s body image and eating disorders [37,38]. A study of male and female Canadian high school students showed that the frequency of appearance comparison with media figures was an even better predictor of body image concerns than the frequency of exposure to thin-ideal media [39]. As SNSs can provide a greater opportunity for social comparison than traditional media by allowing for interpersonal interactions [6], such a negative influence of social comparison might be more easily manifested on SNSs.

Several previous studies have shown that appearance comparison on SNSs plays a role in affecting body esteem. Cohen and Blaszczynski [40], for example, showed that appearance comparison on Facebook (but not on conventional media) led to body dissatisfaction in Australian female students. Ho, Lee, and Liao [41] found that social comparison with friends on SNSs was significantly associated with body image dissatisfaction and the drive to be thin and muscular among Singaporean male and female adolescents, respectively. Another study of male and female students in the United States found a positive association between social comparison frequency on Facebook and the frequency of experiencing negative feelings resulting from this type of comparison [42]. Based on previous research, we thus state the first hypothesis in the following way:

**Hypothesis** **1** **(H1).** *Appearance comparison on Facebook will negatively influence girls’ body esteem.*

### 1.2. Self-Worth Contingency on Others’ Approval

A construct that is relatively new and thus has received only limited focus in the field of body image research is the concept of self-worth contingency. “Contingencies of self-worth represent the domains in which goals are linked to self-worth” [16] (p. 894). In contrast to previous research that mostly evaluated only whether self-esteem is high or low, the concept of self-worth contingency states that individuals differ in the domains on which they base their self-worth. For example, some might focus on academic performance, while others focus on appearance [16,43].

Contingencies of self-worth consist of seven domains: (1) others’ approval; (2) appearance; (3) competition; (4) academic competence; (5) family support; (6) virtue; and (7) God’s love [16]. Among them, the dimensions of appearance and others’ approval are more relevant in the context of body esteem, but we focus in particular on the dimension of others’ approval for several reasons. First, the contingency on others’ approval might be the fundamental reason why adolescents develop an appearance-related self-worth contingency. In contrast to true self-esteem, the views of others are deemed critical for overall contingent self-worth [44], suggesting the importance of the dimension of others’ approval. Additionally, although the correlation between self-worth contingency on appearance and on others’ approval tends to be higher compared to those between other dimensions [16], a study by Overstreet and Quinn [45] reported that the contingency of appearance was not significantly related to body satisfaction, whereas contingency on others’ approval was significant. This finding indicates that while they are closely related, contingency on others’ approval might be a more prominent dimension of self-worth contingency than the contingency of appearance. Second, we focus on others’ approval because it is expected to be sensitive to cultural differences (for a detailed explanation, refer to the following Section 1.3. Cultural Differences). Additionally, while self-worth contingency on appearance has received some academic attention from body image researchers [33,46,47], studies that examined self-worth contingency on others’ approval are rare.

It has been well documented that “[o]thers’ views of the self are an important basis of self-esteem” [48]. Self-esteem has been shown to be related to approval and acceptance from others and what people believe that others think of them [16]. The same view can be assumed for body esteem as a part of self-esteem: Adolescents who are preoccupied with how they are viewed by other people may be less confident about their bodies, as they will assess their own bodies from the perspective of others. Overstreet and Quinn reported that investing self-worth in approval from others was associated with reduced appearance satisfaction [45]. Self-worth contingency on others’ approval was also found to be positively related to body appreciation and eating disorders [49]. Therefore, we propose the following hypothesis:

**Hypothesis** **2** **(H2).** *Girls’ self-worth contingency on others’ approval will negatively relate to body esteem.*

Individuals whose self-worth is based largely on external standards, such as others’ approval, are more likely to seek validation from others, such as in the form of verbal feedback or self-evaluations via social comparison [16,18,44]. This finding has been confirmed in a model on how gratitude, contingencies of self-worth, and social comparison influence body appreciation and intuitive eating, in which a positive relationship between others’ approval and body comparison was found [49]. This is a not particularly surprising finding, as people who seek the approval of others might also be more likely to compare themselves to others, as well as increasingly surveil their own bodies [45,49]. In relation to SNSs, self-worth contingency on others’ approval was found to be linked to Facebook addiction and excessive usage time [43]. This finding might be due to heavy engagement in SNSs with the aim of surveying the views of others and comparing them with one’s own. Therefore, we propose the following hypothesis:

**Hypothesis** **3** **(H3).** *Self-worth contingency on others’ approval will positively influence girls’ appearance comparisons on Facebook.*

### 1.3. Cultural Differences

Research concerning body image has focused primarily on Western cultures. Comparatively few studies have focused on non-Western cultures or have analyzed body image concerns comparatively [26]. Thus, this study makes an important effort in terms of analyzing the relationships between social media use and body image concerns in four cultures (South Korea, Spain, Belgium, and Austria). While South Korea is in East Asia, the other countries are all part of the European Union. Among the many cultural differences in these four cultures, one of Hofstede’s cultural dimensions (individualism versus collectivism) seems to be particularly relevant to the context of social comparison [50]. Hofstede and associates [51] (p. 92) wrote that “individualism pertains to societies in which the ties between individuals are loose: everyone is expected to look after him- or herself and his or her immediate family,” while collectivism “pertains to societies in which people from birth onward are integrated into strong, cohesive in-groups, which throughout people’s lifetime continue to protect them in exchange for unquestioning loyalty.” On this individualism–collectivism dimension, Belgium scores 75, Austria 55, Spain 51, and South Korea 18 (making it the least individualistic, and most collectivistic, culture). Thus, South Korea is clearly less individualistic (and more collectivistic) than the three European cultures.

Previous studies have indicated that people with higher collectivistic scores are more likely to make social comparisons. For example, one study concerning university students in China and the U.S. found that higher collectivism scores were associated with an increased desire to make comparisons [52]. Similarly, a comparative study concerning both European and Asian Canadians found that Asian Canadians sought out more social comparisons [53]. Additionally, Asians and Europeans may differ in terms of self-worth contingency on others’ approval because the concept of self is construed differently and plays different roles in people’s behaviors depending on their culture. According to Markus and Kitayama [17], in individualistic cultures, the self is construed as unique and independent (independent self-construal), whereas in collectivistic cultures, people tend to construe the self in connection to the social context (interdependent self-construal). For Koreans, who are known to have relatively more interdependent self-construal compared to Europeans, self-worth contingency on others’ approval may play a more critical role because the views of others are integral to shaping their own self-concept.

Given this cultural difference, the previously described hypothesized relationships may differ by culture. For example, the relationships between appearance comparison or self-worth contingency on others’ approval and body-esteem might be stronger among South Korean adolescents. As cross-cultural studies concerning self-worth contingency are rare, we formulated the following research question:

**Reserch Question** **1** **(RQ1):** *Will there be any difference in the aforementioned relationships between European countries (Austria, Belgium, and Spain) and South Korea?*

## 2. Methods

### 2.1. Participants

This study utilized data from a large-scale cross-national survey, which was conducted in Austria, Belgium, Spain, and South Korea, to investigate adolescent media uses and their influences on their well-being (The current article uses data that are part of a larger intercultural study project that examines links between media usage and well-being among adolescents in four different countries. More information about the study project can be obtained by sending an email to the corresponding author.). The survey was conducted during the spring of 2017. The investigators in each country respectively obtained approval from the ethical committees of their own host university.

Early and middle adolescents (12–16 years old) were the targets of this study. For this purpose, the host investigators of each country contacted schools in each country until they obtained at least 300 participants. Seven schools in Austria, eleven schools in Belgium, four schools in South Korea, and five schools in Spain agreed to participate. In those schools, students, with their own and parental consent, participated in the study.

### 2.2. Instrument

The questionnaire was initially constructed in English and then translated into the researchers’ own native languages (German, Dutch, Spanish, and Korean). To ensure the equivalencies of the survey questionnaires, the researchers followed the same guidelines (translation, back-translation, and review by other experienced researchers).

In cross-cultural studies, it is important to check whether the measures were reliable and unidimensional across cultures. Therefore, some items were removed to obtain acceptable reliabilities and unidimensionality of the variables. Reliability and exploratory factor analysis were conducted in SPSS for each country separately. The measurement model showed acceptable reliabilities and unidimensionality in all four countries. After this step, composite variables were computed for the main variables that were used for the main analysis. Table 1 provides the reliabilities of the main variables and correlations among the variables.

*Self-worth contingency on others’ approval* was measured by using Crocker et al.’s (2003) self-worth scale. Five items were included in the survey, but only three items were used in the analysis after the confirmatory factor analysis: “I don’t care what other people think of me”, “What others think of me has no effect on what I think about myself”, and “I don’t care if other people have a negative opinion about me”. This variable was measured with a 7-point Likert scale (1 = “Strongly Disagree” to 7 = “Strongly Agree”). 

Measures for *appearance comparison* on Facebook were adapted from the Physical Appearance Comparison Scale by Thompson, Heinberg, and Tantleff [54]. From the original five items, one item was dropped after the factor analysis, and four were used: “When using Facebook, I compare my physical appearance to the physical appearance of others,” “The best way for a person to know if they are overweight or underweight is to compare their figure to the figure of others in Facebook,” “When using Facebook, I compare how I am dressed to how other people are dressed (when I see new photos they share)”, and “When using Facebook, I sometimes compare my figure to the figures of other people (when I see new photos they share)”. All items were scored from 1 = “Never” to 5 = “Always”.

*Body esteem* was measured by utilizing seven appearance dimension items of the body esteem scale for adolescents and adults [55]. Participants were asked to indicate how often they agreed with the following statements ranging from “Never” (1) to “Always” (5): “I am proud of my body”, “I like what I see when I look in the mirror”, “I am satisfied with my weight”, “I really like what I weigh”, “I’m pretty happy about the way I look”, “I have a good body”, and “I’m looking as nice as I’d like to”.

Along with these key variables, media use was measured and included in the analysis as a control variable. Two indicators were utilized to develop a composite measure of media use: time spent on TV and magazines. Participants were asked to indicate how many minutes per day they spent in general on viewing TV and reading magazines (1 = “Never use it to less than 10 min” to 7 = “>6 h”). These two items were averaged. Additionally, body mass index was taken into consideration for the analysis.

### 2.3. Procedure

Students participated in the study during or after school hours depending on the requests by the schools and teachers. Researchers supervised participants taking the survey to ensure that participants could fill out the questionnaires privately and in silence. A total of 981 female adolescents filled out the questionnaire (*M_age_* = 13.46; *SD_age_* = 1.10; age range = 11–16; for a description of the participants, refer to Table 2). 

### 2.4. Data Analysis

For the main analysis, multi-group structural equation modeling (SEM) with indirect effect estimation was used to assess the proposed relationships. Mplus version 8 [56] was used to analyze the data with the robust maximum likelihood (MLR) estimator. Based on recommendations by Hu and Bentler [57], the following criteria were used: (1) Satorra–Bentler chi-square statistics; (2) Bentler’s comparative fit index (CFI); and (3) the root mean square error of approximation (RMSEA). For CFI, values close to or greater than 0.95 indicate a well-fitting model. For the RMSEA, values less than 0.08 indicate a reasonable fit.

## 3. Results

**Figure 1:**
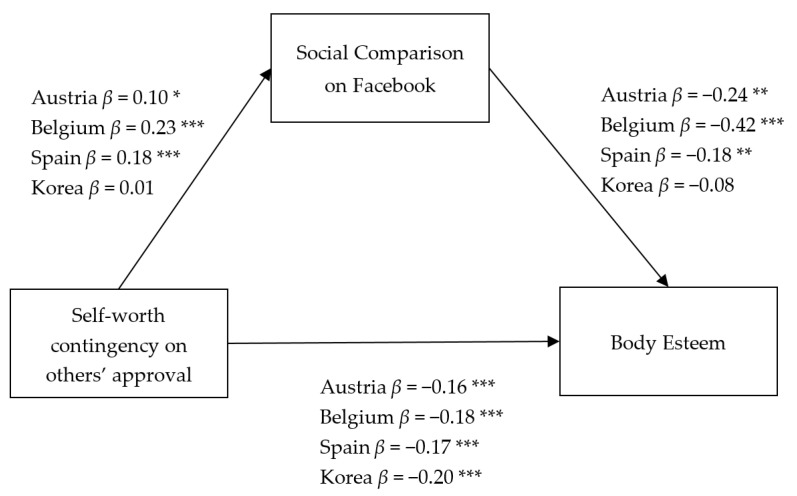
The relationship among self-worth contingency on others’ approval, social comparison on Facebook, and body esteem. * p < 0.05, ** *p* < 0.01, *** *p* < 0.01.

The overall model yielded acceptable goodness-of-fit indices, χ^2^(5) = 5.42, *p* = 0.367, CFI = 0.97, and RMSEA = 0.03. The estimated coefficients of each group are summarized in Figure 1.

The first hypothesis (H1) expected that appearance comparison on Facebook would negatively influence girls’ body esteem. Results consistent with the hypothesis were found with the exception of Korea. As girls engaged more in appearance comparisons on Facebook, they had lower body esteem: Austria (*β* = −0.24, *p* = 0.002), Belgium (*β* = −0.42, *p* = 0.000), and Spain (*β* = −0.18, *p* = 0.003). On the other hand, there was no significant relationship in South Korea (*β* = −0.08, *p* = 0.233).

The second hypothesis (H2) posed that self-worth contingency on others’ approval would negatively influence girls’ body esteem. This hypothesis was supported across the four countries, suggesting that girls whose self-worth is more contingent on others’ approval are more likely to have low body esteem: Austria (*β* = −0.16, *p* = 0.000), Belgium (*β* = −0.18, *p* = 0.000), Spain (*β* = −0.17, *p* = 0.000), and South Korea (*β* = −0.20, *p* = 0.000).

The third hypothesis (H3) expected a positive relationship between self-worth contingency on others’ approval and appearance comparison on Facebook. Similar to the result of the first hypothesis, this hypothesis was supported in all European countries (*β* = 0.10, *p* = 0.01 for Austria; *β* = 0.23, *p* = 0.000 for Belgium; and *β* = 0.18, *p* = 0.000 for Spain). In contrast, it was not supported in South Korea (*β* = 0.01, *p* = 0.86). 

Finally, we posed a research question (RQ1) regarding cultural differences in the hypotheses. As reported above, all three European countries showed the same pattern of relationships, and South Korea was idiosyncratic.

## 4. Discussion

The present study explored the relationships among self-worth contingency on others’ approval, appearance comparisons on Facebook, and body esteem among teenaged girls. This study also examined cultural differences in those relationships by comparing South Korea to European countries (Belgium, Austria, and Spain).

Summarizing the key findings, the first hypothesis regarding the relationship between appearance comparison on Facebook and girls’ body esteem was supported for European girls. Consistent with previous studies [41,42], appearance comparisons on Facebook indeed showed a negative relationship with European girls’ body esteem (whereas this was not the case among Korean girls). This negative relationship might exist because SNS users tend to post edited and polished images of themselves [7,8,9]. As girls are exposed to those images, they naturally engage in social comparison, resulting in lower body esteem. This is in line with studies showing a positive association between body dissatisfaction and appearance-oriented SNS use [12,34,35].

Second, as hypothesized in H2, girls’ self-esteem contingency on others’ approval was negatively related to body esteem among European girls, suggesting that European girls who are more conscious of others are more likely to have lower body esteem. This finding is consistent with previous research which reported that investing self-worth in approval from others is associated with reduced appearance satisfaction [45]. Others’ views of the self are an important basis of self-esteem [48], and it appears that the same applies to body esteem. However, for Korean girls, such a relationship has not been found, as discussed below with regard to cross-cultural differences.

Additionally, consistent with the third hypothesis, girls with higher self-esteem contingency on others’ approval were more likely to engage in appearance comparisons on Facebook, which in turn negatively influenced body esteem. These findings suggest that self-esteem contingency on others’ approval is a critical driver for appearance comparison and low body esteem. This result might be due to the fact that, when people’s self-worth is based largely on external standards, such as others’ approval, they seek validation from others and, thus, engage in greater social comparison [16,18,44].

Finally, in the exploration of RQ1, which concerns cross-cultural differences, we found several differences between South Korea and the European countries. For Korean girls, although self-esteem contingency on others’ approval directly influenced body esteem similar to that seen in European countries, it did not affect appearance comparisons on Facebook. Additionally, appearance comparisons on Facebook were not related to body esteem. One reason for this difference might be the fact that Korean girls are less likely to engage in appearance comparison on Facebook, as the mean score of appearance comparison on Facebook was significantly lower among Korean girls (*M* = 1.70) than among European girls (*M* = 1.96 to 2.02, *p* < 0.05). This lower level of Facebook (comparison) activity might have attenuated its relationship to other variables. Alternatively, this cross-national difference may be attributed to cultural differences; in Korea, which is a more collectivist culture, others’ approval plays a greater role in all parts of life, thus exhibiting a prominent influence over any other variable.

This study has several theoretical implications for body image research on adolescent girls. First, the study adds evidence to the argument that SNSs contribute to body image disturbances by showing that appearance comparison behavior on SNSs exerts a strong negative force on girls’ body-esteem. While some of the previous studies [58] questioned such a relationship, this study is consistent with the majority of the studies as reviewed in Holland and Tiggermann [12]. In particular, our study showed that specific body-related behavior involving social media (appearance comparison on SNSs) is a strong predictor of body disturbance, as suggested in Cohen et al.’s study [30]. Second, this study contributes to body image literature demonstrating the importance of self-worth contingency—the domain of others’ approval. While this concept has received limited attention compared to the domain of appearance, our study shows that self-worth contingency on others’ approval is an important factor for body image-related behaviors and body esteem, given that it directly influences body esteem across all countries and that it also affects appearance comparison behavior on SNSs (in European countries). Finally, taking a cross-cultural approach, this study extends our understanding of the process of body disturbance. It suggests that the effect of body-related SNS use may differ by culture and that theories should be elaborated while taking into account culture-specific factors.

Practical implications for body image intervention programs for girls can be drawn from the findings of this study. Given the negative role of others’ approval of self-worth contingency, educational programs that focus on the importance and value of personal uniqueness might help make adolescent girls less conscious of their appearance. Additionally, SNS literacy programs could play a role, especially in European countries. Such programs may teach adolescent girls to realize that images on SNS tend to be exaggerated and not representative of people’s everyday lives or real-life appearances. In fact, a recent study has shown that social media literacy can protect young adult women from the negative impact of exposure to appearance ideas on social media [59]. Another recent study showed that exposure to “Instagram vs. reality” images containing side-by-side photographs of an idealized depiction and a natural depiction can decrease the detrimental effects of appearance comparison on SNS [60]. Such a campaign could be instrumental to educating adolescent girls.

This study has several limitations that should be examined in future research. First, while participants from three European countries were recruited, Koreans were the only people representing Asia. Therefore, it is unknown whether the findings capture cultural difference (individualism versus collectivism) or country difference. Thus, future studies should include a wider range of cultures to draw more definitive conclusions. Second, given the nature of cross-sectional studies, causal relationships cannot be claimed. It is possible that those who have low body esteem are more likely to engage in appearance comparison on SNSs. Therefore, future studies with experimental or longitudinal research design will be beneficial to examine the causal relationships. For example, studies may test the effect of an intervention program designed to decrease this dimension of self-worth contingency or that have participants engage in appearance comparison behaviors on SNSs. Third, while this study focused on Facebook, social media platforms differ in their natures and the functions they offer. Given the characteristics of each SNS, distinct social media platforms may affect adolescent girls’ body esteem and body-related behaviors differently. For example, Instagram has rapidly gained popularity among teenaged girls and given its image-based contents, Instagram might be a more important source of body dissatisfaction than Facebook. Comparing the effects of different SNS platforms will help to identify how different features of SNSs play a role in the process of body image-related disturbances among adolescent girls.

## 5. Conclusions

Today, a number of studies have shown that SNSs contribute to body image disturbance among young females. Nevertheless, the roles of psychological factors in this process have not received enough attention. In addition, cross-cultural differences have not been thoroughly examined. This study shows that psychological factors such as self-worth contingency on others’ approval are important drivers for appearance comparison on SNSs, which leads to lower body esteem. Using a cross-cultural approach, this study suggests the consequences of body-related SNSs use may differ by culture. These results can be the starting point for intervention programs for girls, such as SNS literacy programs that highlight the exaggerated nature of SNS images and emphasize the importance of personal uniqueness to help girls to be less conscious of the views of others.

## Figures and Tables

**Table 1:** Reliabilities and Correlations.

		Age	BMI	Media Use	Others’ Approval	Social Comparison	Body Esteem
Austria	Age						
	BMI	0.26 **					
	Media Use	−0.08	0.10				
	Others’ Approval	−0.09	0.01	0.04	(0.84)		
	Social Comparison	0.00	0.10	0.19 *	−0.17 **	(0.84)	
	Body Esteem	−0.15 *	−0.35 **	0.02	0.29 **	−0.25 **	(0.89)
Belgium	Age						
	BMI	0.24 **					
	Media Use	0.01	0.09				
	Others’ Approval	0.07	−0.02	0.02	(0.84)		
	Social Comparison	0.21 **	0.21 **	0.06	−0.38 **	(0.81)	
	Body Esteem	−0.09	−0.33 **	0.00	0.39 **	−0.49	(0.89)
Spain	Age						
	BMI	0.09					
	Media Use	0.01	0.03				
	Others’ Approval	0.00	0.06	0.05	(0.83)		
	Social Comparison	0.14 *	0.01	0.16	−0.29	(0.82)	
	Body Esteem	−0.05	−0.22 **	0.02	0.30	−0.23	(0.88)
South Korea	Age						
	BMI	0.16 *					
	Media Use	0.15 *	0.04				
	Others’ Approval	0.02	0.04	0.10	(0.91)		
	Social Comparison	0.34 **	0.10	0.30 *	−0.01	(0.90)	
	Body Esteem	−0.14	−0.20 **	0.18 *	0.37 **	0.04	(0.86)

Note: Reliabilities in parentheses. * p < 0.05, ** *p* < 0.01.

**Table 2:** Demographic Information of Participants.

Variables		Austria (*N* = 199)	Belgium (*N* = 292)	Spain (*N* = 306)	South Korea (*N* = 184)
Age	*M (SD)*	14.11 (1.21)	12.69 (0.67)	14.06 (0.84)	12.96 (0.84)
	Range	12–16	11–15	12–15	12–14
BMI	*M (SD)*	20.41 (3.39)	18.47 (2.94)	20.05 (2.76)	19.90 (2.64)
	Range	14.30–37.04	13.07–31.25	14.06–31.64	14.34–28.35
Media Use	*M (SD)*	4.83 (1.96)	5.02 (1.51)	4.66 (1.84)	5.17 (2.19)
	Range	2–13	2–11	2–12	2–14
Others’ Approval	*M (SD)*	0.18 (0.18)	−0.33 (1.34)	−0.10 (1.42)	−0.72 (1.48)
	Range	−3.49–2.51	−3.16–2.51	−3.49–2.51	−3.49–2.51
Social Comparison	*M (SD)*	0.28 (0.91)	0.23 (0.90)	0.20 (0.89)	−0.05 (0.89)
	Range	−0.76–2.49	−0.76–3.24	−0.76–3.24	−0.76–2.74
Body Esteem	*M (SD)*	3.21 (1.01)	3.27 (0.92)	3.28 (0.92)	2.69 (0.86)
	Range	1–5	1–5	1–5	1–5

## Data Availability

Qualified researchers can obtain the data from the corresponding author (jhchoi@hallym.ac.kr). The data are not publicly available due to privacy concerns imposed by the IRB.
